# Community Transmission of Pneumonic Plague, Fenoarivobe, Madagascar, 2022

**DOI:** 10.3201/eid3211.260549

**Published:** 2026-11

**Authors:** Laurence Randrianasolo, Rivo Rakotoarimisa, Soloandry Rahajandraibe, Aina Harimanana, Nantenaina Ranoelison, Mamy G. Randriamanantsoa, Berthin A. Andrianarisoa, Soanandrasana Rahelinirina, Vaoary Razafimbia, Antso Raherinandrasana, Fanjasoa Rakotomanana, Manuela A. Vololoniaina, Rindra V. Randremanana, Minoarisoa Rajerison

**Affiliations:** Institut Pasteur, Antananarivo, Madagascar (L. Randrianasolo, A. Harimanana, N. Ranoelison, B.A. Andrianarisoa, S. Rahelinirina, F. Rakotomanana, R.V. Randremanana, M. Rajerison); Ministry of Public Health, Antananarivo (R. Rakotoarimisa, S. Rahajandraibe, M.G. Randriamanantsoa, V. Razafimbia, M.A. Vololoniaina); University of Antananarivo, Antananarivo (A. Raherinandrasana)

**Keywords:** pneumonic plague, Yersinia pestis, bacteria, respiratory infections, family cluster, outbreak, rural area, Madagascar

## Abstract

The results of an outbreak investigation of pneumonic plague conducted in the plague-endemic district of Fenoarivobe, Madagascar, revealed a familial cluster of plague. Transmission was aided by intrahousehold contact or gatherings and was amplified by delayed plague treatment, highlighting the need for early diagnosis and rapid initiation of appropriate therapy.

In Madagascar, where plague remains endemic, transmission typically occurs during August–April. Most cases are reported in the central highlands, which are located at an altitude above 800 m (*1*). The predominant clinical form in Madagascar is bubonic plague. However, some outbreaks of pneumonic plague occur every year (*2*). On February 18, 2022, the Bongolava Regional Health Directorate reported 8 suspected deaths of pneumonic plague from the district of Fenoarivobe. We conducted outbreak investigations (protocol study approved by the National Ethics Committee of Biomedical Research [reference no. 068 MSANP/CE] on July 29, 2011) to identify the source, reconstruct the transmission history, and contain the spread of the outbreak. We classified all cases according to the World Health Organization’s international definition of plague cases (*3*).

During January 28–March 1, 2022, we identified 21 suspected plague cases (9 in persons who were alive and 12 in persons who were deceased); all deaths occurred at the community level (i.e., involving multiple households). Case-patients had a median age of 28 years (interquartile range 23–41 years); 11 (52.4%) were women and girls and 10 (47.6%) were men and boys. Twelve persons (11 with pneumonic plague and 1 with initially bubonic plague) had specimen testing at the Central Laboratory for Plague (Institut Pasteur, Antananarivo, Madagascar); 6 had PCR-confirmed plague cases (3 in living persons and 3 in deceased persons), and 1 had a probable plague case. The attack rate was 4.6% (21/45,636 inhabitants), and the overall case-fatality rate was 57.1% (12/21). The outbreak lasted 33 days.

All case-patients had respiratory symptoms, and 1 case-patient had a secondary pneumonic form resulting from neglected bubonic plague. The most common symptoms reported by case-patients were cough (81% [17/21]), chest pain (71.4% [15/21]), bloody sputum (66.7% [14/21]), and fever (61.9% [13/21]). The incubation period varied from 1 to 13 days, and the median disease duration was 3 days (interquartile range 1–9 days). The presumed index case-patient had onset of fever, headache, asthenia, and vomiting on January 28. He sought care at the health center the same day, had malaria diagnosed by rapid test, and received antimalarial treatment. On January 31, he died at home after having severe hemorrhagic symptoms. Thereafter, primary, secondary, and tertiary cases occurred (Figure 1).

**Figure 1:**
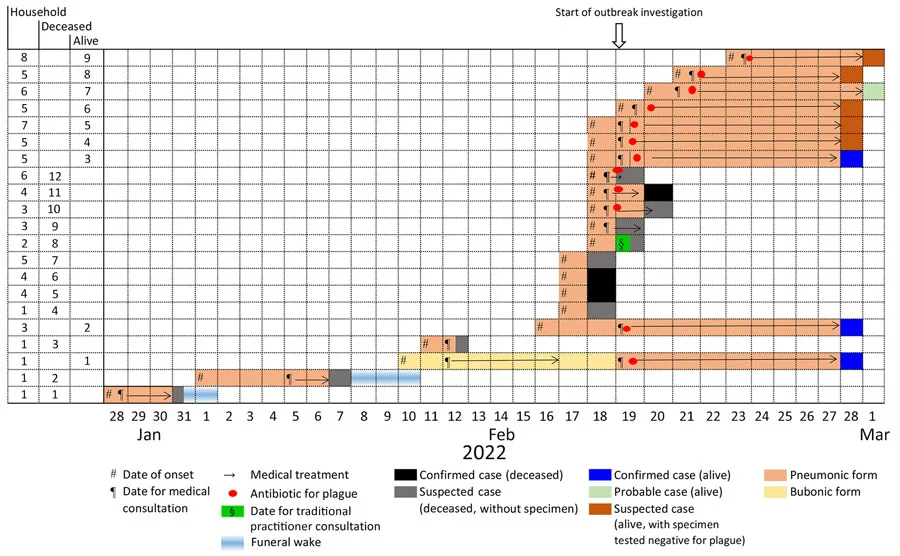
Timeline of events for 21 cases of pneumonic plague in the district of Fenoarivobe, Madagascar, January 28–March 1, 2022.

Twelve case-patients reported having received antibiotics for plague, including the 9 case-patients who were still alive at the time of the outbreak investigation and 3 of 12 deceased case-patients. Anti-plague treatment was administered to case-patients who sought care at health facilities starting in the fourth week of the outbreak, when health workers began to suspect the plague. Among direct contacts of case-patients, 14% (14/100) were positive for F1 IgG based on serologic testing. Identified case-patients were members of 8 households.

This outbreak investigation revealed a familial cluster with a presumed index case-patient who probably had either secondary pneumonic plague occurring after neglected bubonic plague or primary pneumonic plague with an unknown source of contamination. During the first 2 weeks, transmission primarily occurred within the index case-patient’s household, where the primary case-patient was infected by direct contact. Subsequently, more widespread contact occurred at the community level (i.e., with other households) during gatherings (e.g., funerals) or after direct contact with deceased case-patients, based on the transmission routes we reconstructed (Figure 2). 

**Figure 2:**
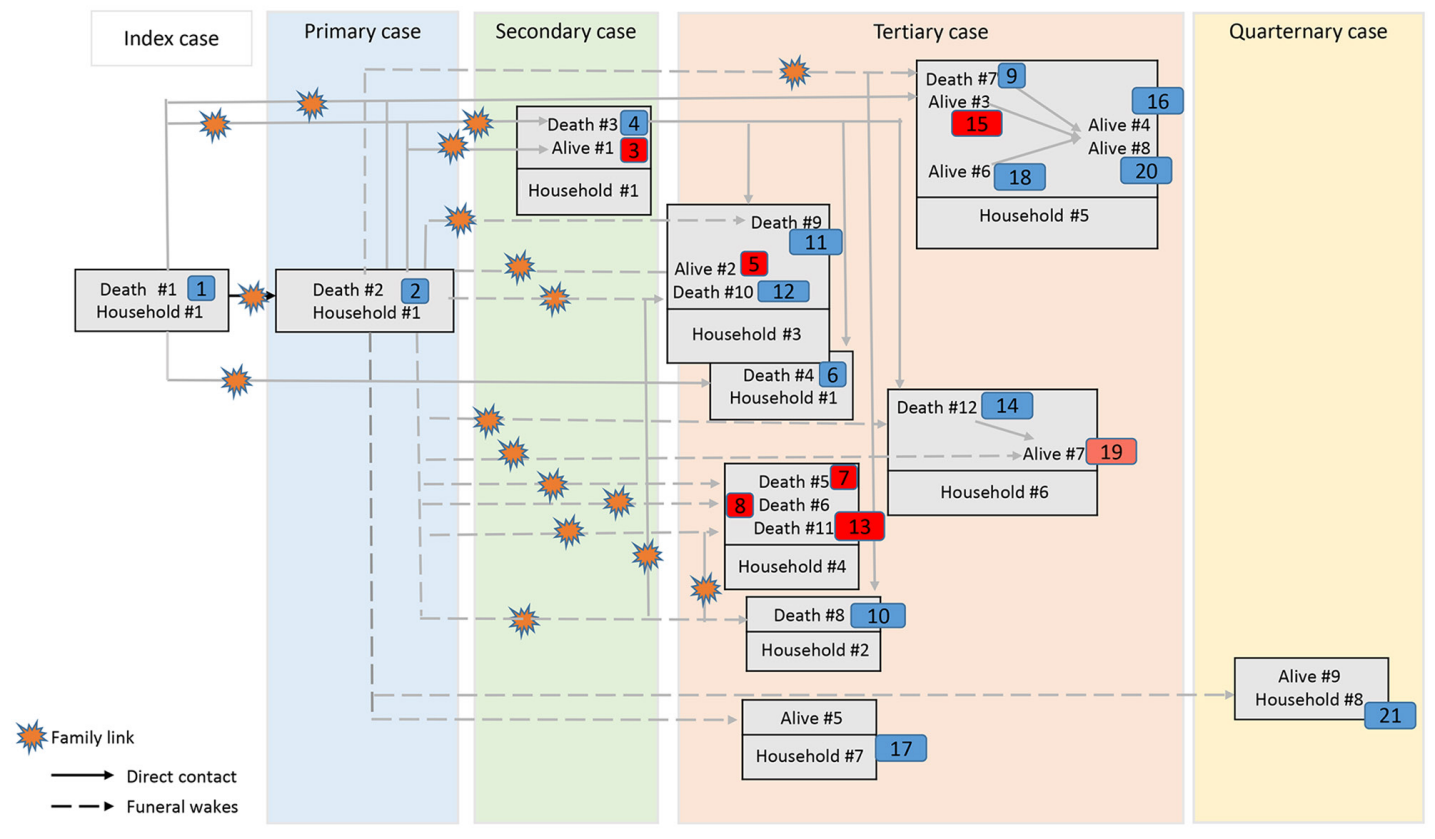
Reconstructed routes of transmission for 21 cases of pneumonic plague in the district of Fenoarivobe, Madagascar, January 28–March 1, 2022. The numbered boxes indicated the order in which cases of plague appeared: confirmed cases are shown in red, probable cases in reddish brown, and suspected cases or those without biological samples in blue.

Early plague treatment is curative, and the high case-fatality rate seemed to be explained by the delay in the initiation of treatment during the first 3 weeks of the outbreak because of misdiagnosis. The outbreak lasted for 1 month, and the appropriate actions to respond to the outbreak were initiated at the beginning of the fourth week, coinciding with our outbreak investigation. Pneumonic plague, like other respiratory diseases, initially manifests with nonspecific symptoms (*4*), and often no clear exposure to infected animals or fleas can be identified, which can lead to misdiagnosis and delay correct management.

This outbreak occurred in a known plague-endemic area. Our serologic results among case-patients suggested that plague continues to circulate in the environment, in the local rodent population, or both, despite the last reported human plague cases occurring 6 years before this outbreak (*5*). Our findings underscore the importance of health workers staying alert in plague-endemic areas to the possibility of reemerging cases of plague and the population to be informed about seeking medical care in case of illness (*6*,*7*).

The transmission dynamics observed in this outbreak highlight the potential for the spread of pneumonic plague, mainly within family and community clusters, particularly in remote settings. Similar patterns of family- and community-based transmission have been described in previous outbreaks in Madagascar, where social structures and caregiving customs contribute to high-risk exposure (*8*–*10*).

Identifying plague and its transmission in remote settings is challenging; therefore, rapid case detection, contact tracing, and timely implementation of preventive measures are essential. Early diagnosis and prompt treatment are important to avoid delays and associated high-fatality rates.
